# Predictors of inadequacy of prenatal tests of postpartum women: a cross-sectional study

**DOI:** 10.1590/0034-7167-2024-0286

**Published:** 2025-09-08

**Authors:** Giovanna Evelyn Luna Silveira, Bruna Barroso de Freitas, Raquel Alves de Oliveira, Victórya Suéllen Maciel Abreu, Bruno Luciano Carneiro Alves de Oliveira, Herla Maria Furtado Jorge, Ana Karina Bezerra Pinheiro, Priscila de Souza Aquino

**Affiliations:** I Federal University of Ceará. Fortaleza, Ceará, Brazil; II Federal University of Maranhão. São Luis, Maranhão, Brazil; III Federal University of Piauí. Teresina, Piauí, Brazil

**Keywords:** Prenatal Care, Nursing, Diagnostic Techniques and Procedures, Prenatal Diagnosis, Public Health, Cuidado Pré-Natal, Enfermagem, Técnicas e Procedimentos Diagnósticos, Diagnóstico Pré-Natal, Saúde Pública, Atención Prenatal, Enfermería, Técnicas y Procedimientos Diagnósticos, Diagnóstico Prenatal, Salud Pública

## Abstract

**Objective:**

to analyze predictors of inadequacy of prenatal care among postpartum women in a maternity hospital in Brazil.

**Methods:**

cross-sectional study conducted from March 2020 to January 2021 with postpartum women from a maternity hospital in Brazil. Statistical analysis was performed using Pearson’s chi-squared test and Poisson regression. All variables with p-values less than 0.2 were included in the regression model.

**Results:**

of the 300 postpartum women, 223 (74.3%) had inadequate prenatal tests, with a higher percentage of inadequate urine tests. The Poisson regression model showed an association between being from the capital city and having more than seven prenatal visits, with a protective effect, RP 0.83 and 0.82, respectively.

**Conclusion:**

access to prenatal care must be ensured for all women in order to improve the performance of tests, early detection of anomalies and treatment of pregnant women, thereby contributing to reducing maternal and neonatal morbimortality.

## RESUMO

**Objetivo:**

analisar preditores de inadequação do pré-natal entre puérperas de uma maternidade no Brasil.

**Método:**

estudo transversal realizado de março de 2020 a janeiro de 2021 com puérperas de uma maternidade no Brasil. Análise estatística foi realizada por meio do teste qui-quadrado de Pearson e regressão de Poisson. Todas as variáveis com valores de p menores que 0,2 foram incluídas no modelo de regressão.

**Resultados:**

das 300 puérperas, 223 (74,3%) apresentaram exames pré-natais inadequados, com maior percentual de exames de urina inadequados. O modelo de regressão de Poisson demonstrou associação entre ser natural da capital e ter realizado mais de sete consultas de pré-natal, com efeito protetor, RP 0,83 e 0,82, respectivamente.

**Conclusão:**

acesso ao pré-natal deve ser garantido a todas as mulheres, a fim de melhorar a realização de exames, a detecção precoce de anomalias e o tratamento das gestantes, contribuindo para a redução da morbimortalidade materna e neonatal.

## RESUMEN

**Objetivo:**

analizar predictores de atención prenatal inadecuada entre puérperas en una maternidad de Brasil.

**Métodos:**

estudio transversal realizado de marzo de 2020 a enero de 2021 con puérperas de una maternidad de Brasil. El análisis estadístico se realizó mediante la prueba de chi-cuadrado de Pearson y la regresión de Poisson. Todas las variables con valores de p inferiores a 0,2 se incluyeron en el modelo de regresión.

**Resultados:**

de las 300 puérperas, 223 (74,3%) tuvieron exámenes prenatales inadecuados, con mayor porcentaje de exámenes de orina inadecuados. El modelo de regresión de Poisson demostró asociación entre haber nacido en la capital y haber tenido más de siete consultas prenatales, con efecto protector, PR 0,83 y 0,82, respectivamente.

**Conclusión:**

se debe garantizar el acceso a la atención prenatal a todas las mujeres, con el fin de mejorar los exámenes, la detección temprana de anomalias y el tratamiento de las gestantes, contribuyendo a la reducción de la morbimortalidad materna y neonatal.

## INTRODUCTION

A positive pregnancy experience is a priority for the World Health Organization (WHO) because of its importance in laying the foundation for healthy motherhood^(1)^. Early initiation and proper management of prenatal care can reduce health inequalities, low birth weight, preterm birth, prevent hypertensive disorders, intervene in cases of severe anemia, and prevent future associated complications^(2)^.

Despite the importance of this care, access to prenatal care remains a significant problem worldwide, influencing outcomes such as high maternal mortality rates^(3)^. This underpins the formulation of the third Sustainable Development Goal, which aims to reduce maternal and child mortality^(4)^.

Indicators of the quality of prenatal care include early initiation of care, referral to specialist services when needed, timely decision-making, and adequacy of routine screening. These tests allow the assessment of a woman’s clinical condition in order to guide appropriate interventions^(5)^.

Studies show that less than 18% of women in Bangladesh received appropriate antenatal care, including access to counselling, testing and receiving results^(6)^. In Paris, a study of 121 homeless women found that 19.3% did not receive adequate antenatal care. Moreover, 9.6% did not receive the appropriate number of tests^(7)^.

In Brazil, this issue is relevant, as research using data from 1,673 users of the Brazilian Unified Health System (In Portuguese, *Sistema Único de Saúde* - SUS), the public health system, found that only 13.2% received all recommended prenatal care^(8)^. Data from the Brazilian Basic Healthcare Information System showed that an average of 60.3% of pregnant women underwent HIV and syphilis testing in 2022^(9)^.

Access to prenatal care and childbirth assistance is influenced by health determinants^(3)^, and it is important to understand them so that healthcare professionals can focus more attention on this population and so that this knowledge can inform women’s health policies. Pregnant women’s adherence to prenatal care, as well as the organization of healthcare services, may be related to adequacy of prenatal care. Therefore, the analysis of predictors of prenatal care attendance is crucial for assessing the services provided by healthcare networks and may highlight fields for improvement in order to facilitate pregnant women’s access to these services. Considering the above, the objective was to analyze predictors of inadequacy of prenatal care among postpartum women in a public maternity hospital in Brazil.

## OBJECTIVE

To analyze predictors of inadequacy of prenatal care among postpartum women in a public maternity hospital in Brazil.

## METHODS

### Ethical considerations

This study was approved by the *Universidade Federal do Ceará* Research Ethics Committee, with approval number 3,673,810, and was conducted in accordance with Resolution 466/12 of the Brazilian National Health Council.

### Study design, period and location

This evaluative and cross-sectional study was conducted from March 2020 to January 2021. The study adhered to the guidelines outlined in the STrengthening the Reporting of OBservational studies in Epidemiology (STROBE) protocol, which were validated specifically for observational research designs^(10)^. Data collection took place at a maternity hospital recognized for obstetric care in Fortaleza, the capital city of Ceará.

### Population, sample and criteria

Postpartum women in the immediate postpartum period, with uncomplicated pregnancies followed up in primary care and with live newborns were included. Postpartum women who had been hospitalized during pregnancy were excluded because hospitalization can compromise continuity of care in primary care, can lead to women missing appointments and routine tests, and there is no guarantee that all hospital tests will be recorded in the pregnant woman’s handbook.

Postpartum women with emotional issues, such as postpartum depression, anxiety, or other psychological disorders, were excluded from this study due to the potential impact of these factors on women’s ability to provide accurate and consistent responses during data collection. These conditions could introduce bias into the results. This approach ensured a more homogeneous and representative analysis of the group of women in good emotional health, enabling a precise assessment of predictors of inadequate prenatal tests by controlling for variables that could distort the data. Thus, the study focused on analyzing predictors of inadequate prenatal tests related to factors more directly associated with the prenatal care process rather than external factors such as postpartum women’s psychological state.

The final sample consisted of 300 women, selected using convenience sampling and sample calculation for a finite population.

### Study protocol

After obtaining informed consent, data were collected directly through bedside interviews in the maternity ward and indirectly through analysis of medical records and the pregnant woman’s handbook. The first part of the questionnaire-based analysis included sociodemographic variables such as age, ethnicity, marital status, place of origin, religion, education, income, and paid employment during pregnancy. Obstetric variables included number of pregnancies, history of miscarriage, difficulty conceiving, and number of living children. Access to prenatal care and quality of care variables included waiting time to schedule an test, time to receive results for the first and second routine tests, prenatal care provider, transportation difficulties, distance to prenatal care facility, distance to test site, gestational age at start of prenatal care, and number of prenatal care visits.

To define the outcome variable, the minimum required tests were identified, which included ABO blood grouping, two hematocrits, two fasting blood glucose tests, two VDRL tests, two anti-HIV tests, two urinalyses, two urine cultures, two HBsAg tests, one toxoplasmosis serology test, one syphilis rapid test, and one HIV rapid test^(11)^.

### Analysis of results, and statistics

Data were tabulated and processed using the Statistical Package for the Social Sciences (SPSS) software. Descriptive analysis of postpartum women’s sociodemographic and obstetric characteristics was performed.

The Shapiro-Wilk test was used to assess the normality of numerical data, while Pearson’s chi-squared test was used to analyze the statistical significance of associations, with prevalence ratio (PR) values calculated using individual raw robust variance. A 5% significance level was used.

A multivariate Poisson regression model with robust variance estimation was performed to estimate PR and 95% Confidence Intervals for variables with a p-value less than 0.2 in Pearson’s chisquared test, resulting in a total of ten variables included in the model.

## RESULTS

The sample consisted of 300 women. Regarding age, 232 (77.3%) women were older than 19 years, with a minimum age of 13 years and a maximum age of 45 years. As for skin color, 226 (75.3%) self-identified as mixed-race. Furthermore, 223 (74.3%) had a partner; 185 (61.7%) had more than nine years of education; and 194 (64.7%) were not employed during pregnancy. Table 1 shows the inadequacy of each mandatory test.

**Table 1:** Distribution of frequency of inadequacy for each recommended routine test among postpartum women surveyed. Ceará, Fortaleza, Brazil, March 2020 to January 2021

Requested tests	Inadequacy	
	n	%
ABO typing	39	13.0
Hematocrit/hemoglobin	138	46.0
Fasting blood glucose	153	51.0
VDRL	161	53.7
Anti-HIV	146	48.7
Urinalysis + urine culture	184	61.3
HBsAg	182	60.7
Serology for toxoplasmosis	44	14.7
Rapid syphilis test	158	52.8
Rapid HIV test	133	44.3
Completion of all mandatory tests	223	74.3

*ABO: Blood type; VDRL: Venereal Disease Research Laboratory; HBsAG: hepatitis B virus surface antigen.*

Considering the minimum number of tests recommended by the Brazilian government for adequacy, as outlined in the methodology of this study, it becomes evident that among the ten mandatory tests, five exhibited inadequacy levels exceeding 50%. In addition, a total of 223 postpartum women (74.3%) showed inadequacy in all the recommended prenatal tests. Table 2 shows the association of sociodemographic and obstetric variables with inadequacy of prenatal care among participants.

**Table 2:** Association of sociodemographic and obstetric variables with inadequacy of tests among interviewed postpartum women. Ceará, Fortaleza, Brazil, March 2020 to January 2021

Variables	Inadequate tests		Adequate tests		*p* value	PR (95%CI)^b^
	n	%	n	%		
Age					0.019^a^	2.35 (1.13-4.88)
Up to 19 years	58	85.3	10	14.7		
Over 19 years	165	71.1	67	28.9		
Place of origin - capital					0.021^a^	2.25 (1.11-4.56)
No	61	84.7	11	15.3		
Yes	162	71.1	66	28.9		
Ethnicity					0.998^a^	1.00 (0.54-1.82)
Mixed-race	168	74.3	58	25.7		
Others	55	74.3	19	25.7		
Marital status					0.007^a^	2.44 (1.26-4.72)
Without partner	74	85.1	13	14.9		
With partner	149	70	64	30		
Religion					0.255^a^	1.43 (0.76-2.68)
No affiliation	162	72.6	61	27.4		
With affiliation	61	79.2	16	20.8		
Education					0.010a	2.11 (1.19-3.75)
Up to 9 years of study	95	82.6	20	17.4		
Over 9 years of study	128	69.2	57	30.8		
Paid occupation during pregnancy					0.015ª	
No	153	78.9	41	21.1		1.91 (1.13-3.25)
Yes	70	66	36	34		
Income					0.047ª	1.69 (1.003-2.87)
Up to 1 minimum wage	119	79.3	31	20.7		
Above 1 minimum wage	104	69.3	46	30.7		
Number of pregnancies					0.673ª	1.12 (0.66-1.90)
Primiparous	93	75.6	30	24.4		
Multiparous	130	73.4	47	26.6		
Abortions					0.334ª	1.40 (0.70-2.82)
Yes	31	79.5	8	20.5		
No	192	73.6	69	26.4		
Difficulty becoming pregnant					0.842ª	1.08 (0.48-2.41)
No	195	74.1	68	25.9		
Yes	28	75.7	9	24.3		
Number of living children					0.652ª	1.12 (0.67-1.89)
1	108	75.5	35	24.5		
2 or more	115	73.2	42	26.8		

*Note:^a^ Pearson’s chi-square test;^b^ Raw Prevalence Ratio with robust variance; CI – Confidence Interval.*

Statistical significance was found by Pearson’s chi-squared test in association with age up to 19 years, rural origin, absence of a partner, education up to 9 years, absence of paid employment, and income up to 1 minimum wage, with a PR greater than 1 for all associations. Obstetric variables did not show statistical significance with inadequacy of tests. Table 3 shows the association of access and quality of prenatal care variables with inadequacy of tests among the interviewed postpartum women.

**Table 3:** Association of access and quality of prenatal care variables with inadequacy of tests among interviewed postpartum women. Ceará, Fortaleza, Brazil, March 2020 to January 2021

Variables	Inadequate tests		Adequate tests		*p* value	PR (95%CI)^d^
	n		n	%		
Delay in scheduling appointments					0.421^a^	0.75 (0.37-1.51)
Yes	44	78.6	12	21.4		
No	179	73.4	65	26.6		
Time to receive 1^st^ routine tests					0.033^a^	3.05 (1.04-8.94)
Less than or equal to 30 days	32	88.9	4	11.1		
Greater than 30 days	191	72.3	73	27.7		
Time to receive 2^nd^ routine tests					0.014^a^	4.13 (1.22-13.90)
Less than or equal to 30 days	32	72.1	3	27.9		
Greater than 30 days	191	72	74	28		
Difficulty moving around					0.290^a^	1.55 (0.68-3.51)
Yes	34	81	8	19		
No	189	73.3	69	26.7		
Distant prenatal service^c^					0.960^a^	0.98 (0.57-1.70)
No	147	74.2	51	25.8		
Yes	76	74.5	26	25.5		
Remote test location					0.937^a^	1.02 (0.60-1.71)
Yes	117	74.5	40	25.5		
No	106	74.1	37	25.9		
GA^b^ at the beginning of PC^c^					0.047^a^	1.69 (1.006-2.87)
Above 12 weeks	122	79.2	32	20.8		
Up to 12 weeks	101	69.2	45	30.8		
Number of PC^c^ appointments					0.01^a^	2.51 (1.45-4.33)
Up to 7	122	86.7	25	13.3		
Above 7	101	67.7	52	32.3		

*Note:^a^ Pearson’s chi-square test;^b^ Gestational age;^c^ Prenatal care;^d^ Raw prevalence ratio with robust variance.*

As shown in Table 3, Pearson’s chi-squared test revealed an association between time to first and second routine tests, initiation of prenatal care after the 12^th^ week of pregnancy and having up to seven prenatal care visits with the occurrence of prenatal inadequacy. Table 4 shows the Poisson regression with robust variance of variables with a p-value less than 0.2.

**Table 4:** Poisson regression with robust variance. Ceará, Fortaleza, Brazil, March 2020 to January 2021

Variables	P value	APR^a^	95%CI^b^
Age up to 19 years	0.265	0.92	0.81-1.05
Age above 19 years	-	1	-
Capital city of the state	0.007	0.83	0.73-0.95
Countryside	-	1	-
With a partner	0.049	0.87	0.77-1.00
Without a partner	-	1	-
Education up to 9 years	0.197	0.91	0.80-1.04
Education above 9 years	-	1	-
Paid occupation	0.220	0.90	0.77-1.06
Unpaid occupation	-	1	-
Income up to 1 minimum wage	0.341	0.93	0.82-1.06
Income above 1 minimum wage	-	1	-
Receipt of first tests greater than 30 days	0.818	1.02	0.80-1.30
Receipt of first tests less than 30 days	-	1	-
Receipt of last tests greater than 30 days	0.140	1.17	0.94-1.46
Receipt of last tests less than 30 days	-	1	-
Initiation of PC^c^ after 12 weeks	0.599	0.96	0.83-1.10
Initiation of PC^c^ before 12 weeks	-	1	-
Number of appointments above 7	0.004	0.82	0.72-0.94
Number of appointments up to 7	-	1	-

*Note:^a^ Adjusted prevalence ratio;^b^ Confidence Interval;^c^ Prenatal care.*

Poisson regression with robust variance showed that women from the capital city had a 17% lower prevalence of inadequate prenatal laboratory testing, while women with more than seven prenatal visits had an 18% lower prevalence of inadequate prenatal testing. These were the only variables that remained associated.

## DISCUSSION

It was evident that inadequacy of prenatal care among pregnant women was associated with being from rural areas, not having a partner, having up to nine years of education, not having paid employment, having an income of up to 1 minimum wage, and having a lower number of appointments. Inadequacy of prenatal care among pregnant women up to 19 years of age is consistent with studies showing that older pregnant women receive more adequate prenatal care^(12, 13)^ and that sociodemographic factors influence women’s prenatal care^(14)^.

Socioeconomic characteristics and prenatal care received were more favorable for non-adolescent mothers, as adolescents had fewer prenatal appointments and delayed initiation of these appointments, which hinders better prenatal care and consequently leads to low adherence to recommended tests^(15)^. A study showed that the risk of severe maternal morbidity was 1.8 times higher among adolescent mothers who received inadequate prenatal care^(16)^.

From this perspective, the importance of prenatal care in conducting tests and screening for complications is emphasized, as evidenced by a study conducted in Peru. Inadequate prenatal care is associated with a higher incidence of newborns with macrosomia and low weight for gestational age, which could be detected by the tests performed during prenatal care^(17)^.

Regarding the presence of a partner, women who had a partner were less likely to have inadequate laboratory tests. Research shows that 76.5% of married women in labor received more adequate prenatal care. This finding may be related to the importance of family support in encouraging care^(18)^. In the United States, a positive association was found between marriage and prenatal care, as marriage was associated with a lower likelihood of late initiation or no prenatal care^(19)^.

The level of knowledge and education influences women to seek services and care for their health status^(20)^. Pregnant women with 12 or more years of education showed a higher proportion of prenatal care visits, emphasizing that maternal education level influences prenatal care attendance^(21)^. In the present study, women with up to nine years of education had a higher prevalence of inadequate laboratory tests, confirming the findings of previous studies.

The association of lack of paid employment and income up to one minimum wage with inadequate testing in this study is consistent with research from the United States, which found that low-wage pregnant women often have limited flexibility in working hours and paid leave, making it even more difficult to keep prenatal appointments^(22)^. In Brazil, a study conducted in Paraná found that women, even those with paid employment, did not give up access to prenatal care by exercising their labor rights to take time off for these appointments^(23)^.

The inadequacy of the tests observed in 74.3% of the women in this study is consistent with the results of a study conducted in Canada, which found an inadequacy rate of 11.6%, with 84.4% classified as intermediate. This classification considered negative health outcomes resulting from the number of appointments and interventions during prenatal care^(24)^. These data show that the problem of inadequacy is not unique to Brazil, underscoring the need to investigate vulnerabilities related to inadequacy in prenatal care in order to prevent negative health outcomes for both mother and child.

In New York, evidence-based recommendations were published to promote universal screening and diagnosis of HIV in all pregnant women and women in labor. These guidelines aimed to prevent perinatal transmission and protect the health of mothers and babies^(25)^. In Brazil, 25.4% of pregnant women underwent all the tests recommended by the Ministry of Health, a finding similar to the present study, in which only 25.7% achieved an adequate level of testing^(26)^. It was also evident that 9.2% of pregnant women reported lack of access to the recommended third trimester tests: urine analysis, blood glucose testing, and laboratory screening for sexually transmitted infections^(27)^.

In Canada, despite having a universal healthcare system, 11.5% of women experienced inadequate prenatal care. Among the factors associated with inadequate prenatal care, living in the North or in a rural area stood out^(13)^. This finding is consistent with the results of this study, as it highlights that inadequate care is associated with being from rural areas of the state and having fewer appointments.

The analysis of the origin of pregnant women is essential, considering that pregnant immigrant women without health insurance in developed countries often receive inadequate prenatal care^(28)^, which reinforces the need to inquire about the origin of these women in order to ensure continuity of care.

The prevalence of prenatal care in this study was 62.8%. In Ethiopia, the prevalence of prenatal care use was considered low, with factors positively associated with use including higher maternal education, frequent radio listening, higher wealth status, traditional beliefs, and larger number of children^(29)^. This suggests that education, income and number of children act as protective factors for prenatal care use, as higher education is associated with awareness of the benefits of prenatal care at this stage in a woman’s life.

In terms of prenatal care visits, this study identified inadequate care, with most women initiating prenatal care after 12 weeks of gestation and attending up to seven visits. Conversely, in Africa, it was observed that despite pregnant women initiating prenatal care early, they did not undergo all the necessary tests^(30)^.

In East Africa, inadequate completion of testing was observed, with 4.5% of pregnant women not being tested for HIV, 8.8% for syphilis, 39.9% for hepatitis B, 25.8% for malaria, 25.5% for hemoglobin, 45.4% for glucose, 29.7% for urine, and 41.1% for ultrasound. Reasons for non-completion included lack of financial support to afford tests and lack of reagents available at health facilities^(30)^.

A descriptive cross-sectional study in the state of Ceará, using data from the Prenatal Care Monitoring System, revealed a decrease in the completion rate of tests. Initially, 45% of women underwent essential laboratory tests, including blood, hemoglobin (Hb), hematocrit (Ht), HIV, and initial urine, glucose, and sexually transmitted infection tests during the first trimester. However, the percentage significantly decreased when compared to women who had testing in the 1^st^ and 3^rd^ trimesters, with the majority (29.2%) lacking access to the second urine, glucose, and sexually transmitted infection tests^(27)^.

Inadequacy of prenatal screening highlighted in this study undermines overall quality of prenatal care. Completing the tests helps to identify and treat preexisting conditions, reducing pregnancy and childbirth complications, and lowering the risk of premature births and miscarriages. Additionally, screening results empower healthcare professionals to offer more effective clinical and nutritional guidance to pregnant women.

Variations in quality of prenatal care and associated factors may arise from differences in equipment availability, infrastructure, workload, and commitment among prenatal care providers, leading to inconsistencies in maternal health information^(31)^. Pregnant women often seek care in environments that are welcoming and patient-centered^(32)^. Our study reinforces this observation by showing, through Poisson regression analysis, that inadequate test completion is linked to rural residence and fewer appointments.

Ensuring access to prenatal care is crucial for maternal and child health outcomes. Therefore, facilitating this access is a valid and vital strategy to reduce maternal and child morbidity and mortality rates. One potential solution to address the challenge of reaching women living far from health centers is the implementation of mobile clinics for prenatal appointments. For instance, in Jordan, pregnant women received prenatal care through mobile clinics, demonstrating the feasibility of this approach in providing care to those in remote areas with limited economic resources, considering their vulnerabilities^(33)^.

Based on the above, it appears that deficiencies in this group may stem from delays in completing and receiving laboratory tests as well as from fewer appointments and delayed initiation of prenatal care. Challenges in obtaining results and pregnant women giving up due to obstacles encountered could lead to a low prevalence of timely receipt, consequently contributing to inadequate prenatal care.

### Study limitations

Limitations of this study include its single-site nature, which precludes the provision of data from other regions. Moreover, the paucity of studies specifically reporting on inadequacy of prenatal screening contributed to a broader discussion about prenatal care as a whole rather than detailing the outcome, highlighting the need for further in-depth studies specifically on this topic.

### Contributions to nursing and public health

It is important to emphasize the need for further research in nursing, as nurses play a crucial role in prenatal care. Based on the findings of this study, they can explore ways to improve pregnant women’s adherence to prenatal care and, consequently, to screening processes. Finally, there is a need for studies in this field that assess healthcare professionals’ knowledge, attitudes, and practices, as these are critical aspects in achieving optimal outcomes.

## CONCLUSION

In conclusion, this study shows that the lack of screening is intricately linked to overall prenatal care and is influenced by various sociodemographic and obstetric factors, as well as by the postpartum woman herself, her access to services, and the healthcare professionals involved. These factors may influence adequacy of prenatal care in different ways.

Predictors of inadequacy included being younger than 19 years, living in a non-urban area, having less than nine years of education, being out of paid employment during pregnancy, having an income below the minimum wage, starting prenatal care later than 12 weeks of pregnancy, having fewer than seven prenatal visits, and waiting more than 30 days between the first and subsequent routine visits. In addition to this, Poisson regression analysis revealed an association between urban residence, receiving prenatal care from a nurse, and having up to seven prenatal visits with inadequate prenatal care.

## Data Availability

Not applicable.
